# Hand-Assisted laparoscopic donor nephrectomy PERiumbilical versus Pfannenstiel incision and return to normal physical ACTivity (HAPERPACT): study protocol for a randomized controlled trial

**DOI:** 10.1186/s13063-018-2775-4

**Published:** 2018-07-13

**Authors:** Yakup Kulu, Beat P. Müller-Stich, Omid Ghamarnejad, Elias Khajeh, Georgios Polychronidis, Mohammad Golriz, Felix Nickel, Laura Benner, Philipp Knebel, Markus Diener, Christian Morath, Martin Zeier, Markus W. Büchler, Arianeb Mehrabi

**Affiliations:** 1 Department of General, Visceral, and Transplantation Surgery, University of Heidelberg, Im Neuenheimer Feld, 110 69120 Heidelberg, Germany; 2 Department of Nephrology, Heidelberg University Hospital, Heidelberg, Germany; 3 Institute of Medical Biometry and Informatics, University of Heidelberg, Heidelberg, Germany

**Keywords:** Living donor, Hand-assisted laparoscopy, Physical activity, Randomized clinical trial

## Abstract

**Background:**

Hand-assisted laparoscopic living donor nephrectomy (HALDN) using a periumbilical or Pfannenstiel incision was developed to improve donor outcome after a kidney transplant. The aim of this study was to investigate two methods of hand assistance and kidney removal during HALDN and their effect on the time it takes for the donor to return to normal physical activity.

**Methods/design:**

This study was initiated in November 2017 and is expected to last for 2 years. To be eligible for the study, donors must be more than 20 years of age and must not be receiving permanent pain therapy. Only donors with a single artery and vein in the graft are being enrolled in this trial. Donors with infections or scars in the periumbilical or hypogastric area, bleeding disorders, chronic use of immunosuppressive agents, or active infection will be excluded. Donors will be randomly allocated to either a control arm (periumbilical incision) or an intervention arm (Pfannenstiel incision). The sample size was calculated as 26 organ donors in each group. The primary endpoint is the number of days it takes the donor to return to normal physical activity (up to 4 weeks after the operation). Secondary endpoints are intraoperative outcomes, including estimated blood loss, warm ischemia time, and duration of the operation. Postoperative pain will be assessed using the visual analog scale, rescue analgesic use, and peak expiratory flow rate. Length of hospital stay, physical activity score, time to return to work, donor satisfaction, cosmetic score, postoperative complications, and all-cause mortality in living donors will also be reported. Delayed graft function, primary non-function, serum creatinine levels, and glomerular filtration rate will also be assessed in the recipients after transplantation.

**Discussion:**

This is the first randomized controlled trial to compare the time it takes the living donor to return to normal physical activity after HALDN using two different types of incision. The comprehensive findings of this study will help decide which nephrectomy procedure is best for living donors with regard to patient comfort and satisfaction as well as graft function in the recipient after transplantation.

**Trial registration:**

ClinicalTrials.gov, NCT03317184. Registered on 23 October 2017.

**Electronic supplementary material:**

The online version of this article (10.1186/s13063-018-2775-4) contains supplementary material, which is available to authorized users.

## Background

Despite remarkable improvements in kidney procurement from deceased donors, the demand for organs is still higher than the number of available kidneys. The use of living donor kidney transplantation (LDKT) has been increased in an attempt to overcome the organ shortage [1–4]. The main concern of LDKT is that a healthy person undergoes major surgery to supply an organ, which may have serious adverse effects. Therefore, a safe nephrectomy method with a low complication rate is just as important as ensuring good graft quality after transplantation [3, 5].

Open donor nephrectomy was the most common technique for kidney removal before laparoscopic surgery was developed [6, 7]. Laparoscopic nephrectomy has obvious intraoperative and postoperative advantages over open surgery in terms of blood loss, postoperative pain, duration of hospital stay, and convalescence [8–10]. In 1998, shortly after the development of commercial ports, Wolf et al. [11] reported the hand-assisted laparoscopic living donor nephrectomy (HALDN) technique. Since then, this technique has been widely adopted [12]. HALDN offers better manual control of bleeding, a shorter learning curve, less kidney traction, faster kidney extraction, and shorter warm ischemia times [7, 11–13]. Nowadays, HALDN is one of the most commonly applied surgical procedures, and it has a relatively low complication rate [13].

HALDN is usually performed using a periumbilical or Pfannenstiel incision for hand-port placement and kidney extraction [14]. So far, several retrospective and non-randomized prospective studies have compared periumbilical and Pfannenstiel incisions [13, 15–18]. While some studies have shown better donor outcomes for periumbilical incision with regard to blood loss, warm ischemia time, duration of the surgery, and postoperative pain [15, 17, 18], Pfannenstiel incision is associated with fewer early and long-term wound complications and better cosmetic outcomes [13, 16]. Donors were also able to return to work more quickly after a Pfannenstiel incision [15]. However, these conclusions were the results of retrospective or non-randomized prospective studies. To our knowledge, the use of these two different incisions during HALDN has not been compared in a randomized controlled trial (RCT).

In this RCT, we are investigating the effect of two different hand-assistance and kidney removal techniques during HALDN, using periumbilical and Pfannenstiel incisions, on the time it takes for donors to return to normal physical activity. We will also analyze graft function in the recipient after transplantation.

## Methods/design

### Setting

This is a single-center, expert-based RCT. The trial is now underway at the Division of Transplantation Surgery, Department of General, Visceral, and Transplantation Surgery, University of Heidelberg. It was initiated in November 2017, and it is expected to progress for 2 years. The trial was registered at ClinicalTrials.gov under registration number NCT03317184 on October 23, 2017. The Standard Protocol Items: Recommendations for Interventional Trials (SPIRIT) checklist is provided in Additional file 1.

### Patient recruitment

**Fig. 1:**
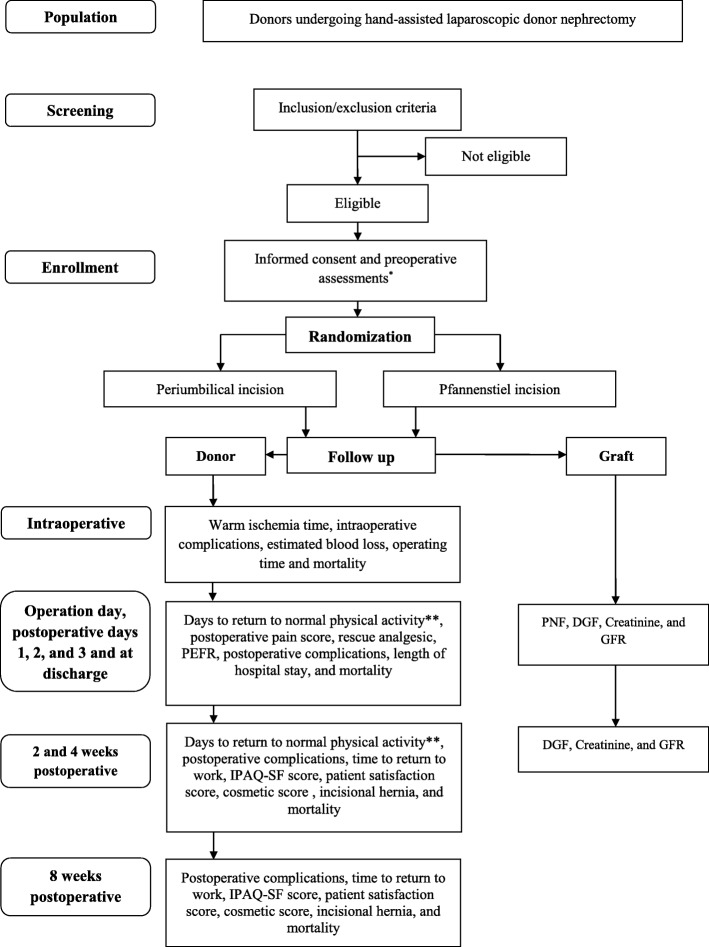
Study design flow chart. *Preoperative assessments including date of birth, gender, weight (kg), height (cm), and American Society of Anesthesiologists score. **Return to normal physical activity will be self-assessed using the Katz Basic Activities of Daily Living self-maintenance questionnaire during the first 4 postoperative weeks until all normal activities are recovered. *PEFR* peak expiratory flow rate, *PNF* primary non-function, *DGF* delayed graft function, *GFR* glomerular filtration rate, *IPAQ-SF* International Physical Activity Questionnaire-Short Form

The study protocol was accepted by the independent Ethics Committee of the University of Heidelberg (registration number S-291/2017). As shown in the study flow chart (Fig. 1), all kidney donors (including left and right kidneys) are currently being screened for eligibility criteria. To be eligible for the study, living donors must be more than 20 years of age and must not be receiving permanent pain therapy. Only donors with a single artery and vein in the graft are being enrolled in this trial. Donors with infections or scars in the periumbilical or hypogastric area, bleeding disorders, chronic use of immunosuppressive agents, and active infection are being excluded. All included donors will be informed about periumbilical and Pfannenstiel incisions for HALDN, as well as their potential benefits and side effects. Eligible donors will receive a written informed consent form. Donors who sign the informed consent form will be included in the study. Reasons for exclusion from the Hand-Assisted laparoscopic donor nephrectomy PERiumbilical versus Pfannenstiel incision and return to normal physical ACTivity (HAPERPACT) trial will be documented and explained in the screening form. After baseline assessments and assessment of eligibility, the patient will be randomized to the periumbilical or Pfannenstiel arm.

### Outcome measures

**Fig. 2:**
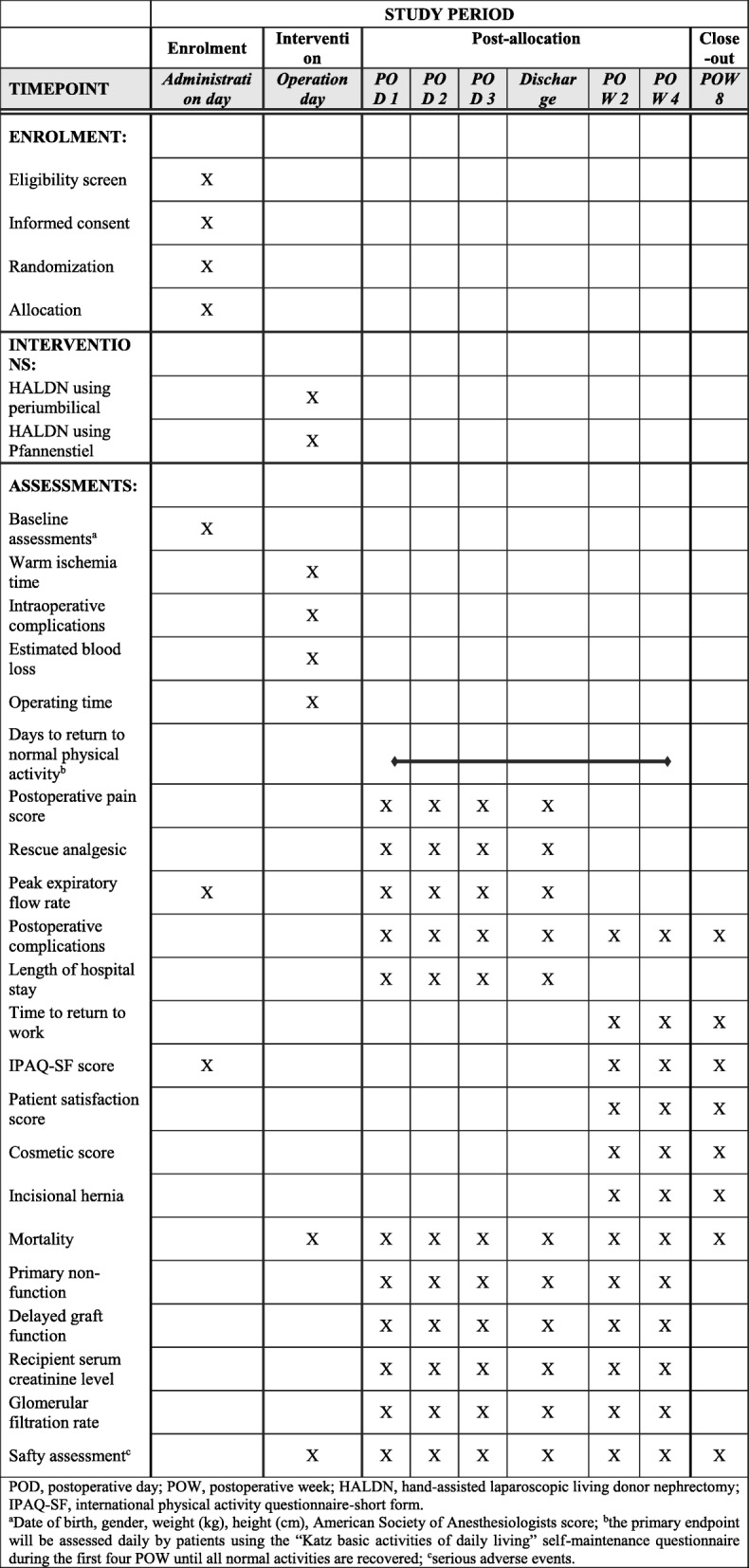
HAPERPACT trial design according to the Standard Protocol Items: Recommendations for Interventional Trials (SPIRIT) checklist

During the HAPERPACT trial, donors will be monitored before surgery, intraoperatively, on postoperative days (PODs) 1, 2, 3, and at discharge. After discharge, patients will be visited during postoperative weeks (POWs) 2, 4, and 8. During the first four POWs, donors will complete a self-administered questionnaire every day. Demographic and baseline clinical data, intraoperative findings, and postoperative results will be recorded (Fig. 2). Furthermore, graft function will be assessed until the fourth POW.

To enhance participant retention and to avoid loss to follow-up, we call the patients during the follow-up period to remind them of scheduled visits and to arrange appointments. When a patient is not able to participate in a follow-up visit, we will speak with the patient on the telephone and ask him/her to send us a photo of the incision.

#### Primary endpoint

Patients will complete the Katz Basic Activities of Daily Living (ADL) self-maintenance questionnaire each day during the first four POWs until all normal activities are recovered. This questionnaire indicates their ability to perform daily living activities [19]. The Katz ADL evaluates the patient’s independent performance in six functions: bathing, dressing, using the toilet, transferring, continence, and feeding. These functions are evaluated with yes/no questions. A score of 6 indicates independence in all six functions, demonstrating normal physical activity. Since all donors are able to perform these basic activities before the operation, the Katz ADL questionnaires are not administered prior to surgery. The questionnaires will be gathered at each follow-up visit, and the number of days it takes to return to normal physical activity will be determined based on the answers. The primary endpoint has been defined as the number of days from surgery until return to normal physical activity.

#### Secondary endpoints

Intraoperative outcomes, including estimated blood loss, warm ischemia time, and operating time, will be reported. In addition to the Katz ADL questionnaire, a physical activity score will be determined using the International Physical Activity Questionnaire-Short Form (IPAQ-SF). The IPAQ-SF score will be calculated before the operation and at POWs 2, 4, and 8. Postoperative pain will be assessed using the visual analog scale (VAS 0–10, 0 = no pain and 10 = unbearable distress) and by measuring in-hospital rescue analgesics until discharge. The peak expiratory flow rate (PEFR) will also be evaluated preoperatively and until discharge as an objective surrogate measure of pain. Length of hospital stay, time to return to work, postoperative complications, and all-cause mortality will also be reported in living donors. The appearance of incision scars will be evaluated with a patient satisfaction score (Likert scale 1–5, with 5 indicating strongly satisfied and 1 indicating strongly unsatisfied) and a cosmetic score (defined by the Stony Brook Scar Evaluation Scale [20]) during the first eight POWs. Possible incisional hernia at the site of the surgical incision will be examined by palpation and ultrasonography during each visit. The quality of the procured graft will also be evaluated in recipients after both procedures. Delayed graft function, primary non-function, serum creatinine levels, and glomerular filtration rates will be assessed in recipients until POW 4. Secondary outcome measures are defined in Table 1.

**Table 1:** Secondary endpoints of the HAPERPACT trial

Secondary endpoints	Definitions
Warm ischemia time	From the time of clamping the first renal artery in situ to flushing the kidney with chilled solution on the back table (minutes)
Intraoperative complications	Any complication occurring during the operation
Estimated blood loss	The entire blood loss from skin incision to skin closure (milliliters)
Operating time	From the time of skin incision to closure of the skin incision (minutes)
Postoperative pain	Severity of pain measured by the 11-point visual analog scale (0 = no pain and 10 = unbearable distress)
Rescue analgesic	Total amount of analgesics required during the first three postoperative days
Peak expiratory flow rate (PEFR)	Maximum speed of expiration, measured with a peak flow meter
Postoperative complications	Postoperative surgical complications (i.e., burst abdomen and hernia, wound infection, seroma, and hematoma, intra-abdominal bleeding/hematoma, intra-abdominal abscess or collection, lymphocele, and postoperative ileus) and medical complications (pneumonia, pleural effusion, urinary tract infection). Each complication will be graded according to the Clavien-Dindo classification [29]
Length of hospital stay	From the day of the operation until the day of discharge (days)
Time to return to work	The number of days from discharge to return to work
IPAQ-SF score*	The physical activity of patients will be evaluated preoperatively, and on postoperative weeks 1, 2, 4, and 8 using the IPAQ-SF [18]
Patient satisfaction score	Patient satisfaction score measured by the 5-point Likert scale (5 = strongly satisfied and 1 = strongly unsatisfied)
Cosmetic score	Cosmetic score as defined by the Stony Brook Scar Evaluation Scale [20]
Incisional hernia	Fascia or muscle defect (bulging hernial sac and palpable fascia gap) at the site of the surgical incision examined by palpation and ultrasonography
Mortality	Death due to any cause at any time during the follow-up period
Primary non-function (PNF)	The graft never functions
Delayed graft function (DGF)	The need for one or more hemodialysis treatments following transplantation prior to the onset of graft function. The duration of DGF will be calculated from the date of transplantation to the date of the last dialysis treatment
Recipient serum creatinine level	Serum creatinine level (mg/dL)
Glomerular filtration rate (GFR)	GFR (mL/min/1.73 m^2^) calculated as 175 × (S_cr_)^–1.154^ × (Age)^–0.203^ × (0.742 if female)

*IPAQ-SF International Physical Activity Questionnaire-Short Form

### Standardized therapy and trial interventions

The site of hand-port placement will be randomized. All other intraoperative and perioperative treatments are standardized procedures. Prophylactic antibiotic therapy and use of intravenous or oral analgesic agents to relieve postoperative pain will be administered according to our kidney transplantation manual [21].

According to the randomization arm, hand-port placement and hand insertion will be via a midline periumbilical or Pfannenstiel incision. HALDN with a periumbilical incision will be performed by positioning the donor in a left/right lateral decubitus position. A 5-mm laparoscope port will be placed at the midclavicular line in the left/right upper quadrant (two finger breadths below the costal margin), and one working instrument (12 mm) will be placed at the level of the umbilicus to the left/right of the midline. HALDN with a Pfannenstiel incision will be performed by placing the donor in the French position. A four-port transperitoneal approach will be used: a 10-mm umbilical trocar for the laparoscope, one 5-mm port in the midline, one 12-mm port at the level of the umbilicus to the left/right of the midline, and an additional 5-mm port will be inserted at the left/right border of the Pfannenstiel incision. As soon as the blood vessels are divided and the nephrectomy is completed, the graft will be extracted using the hand port. At the end of the operation, an abdominal drain will be placed and the wound will be closed.

### Modification of the protocol

Protocol amendments will be considered by the principal investigator after feedback from the Clinical Trial Center. All protocol amendments will be submitted to the Ethics Committee for approval. No further recruitments will take place until the modifications are accepted.

### Assessment of safety and termination criteria

To assess the safety of the periumbilical and Pfannenstiel approaches, serious adverse events (SAEs) will be observed and evaluated. Only intervention-related events that occur during surgery and follow-up will be documented. At each visit, the physician will ask the donor if he/she has suffered from any SAEs since the last visit. The attending physician must tell the principal investigator about any reported SAEs within 24 h. The report will be complete and include details of the SAE and whether the SAE was caused by the trial treatment. All intervention-related SAEs will be documented and analyzed.

Donors will be excluded from the study if they withdraw their consent to participate in the trial. A donor may withdraw consent at any time without explanation and without affecting further medical care. The principal investigator may terminate the trial at any time in consultation with the key research associates and the biostatistician. Possible reasons for termination include high morbidity or mortality rates and any indication of potential health hazards caused by either the study treatment or external factors.

### Randomization and blinding

Donors will be randomized to the different groups using the block randomization method. Each block contains two groups of periumbilical and Pfannenstiel incisions. The block size is hidden from the trial executers and clinicians. Possible balanced combinations of these groups within the block are numbered consecutively. Then, blocks are randomly chosen using simple randomization software (Microsoft Excel®), and a series of randomly assigned periumbilical and Pfannenstiel incisions are generated based on the random sequence of blocks. Allocation will be concealed using sequentially numbered, sealed opaque envelopes prepared by a member of our Clinical Trial Center. Donors will be randomly allocated to either the control arm (periumbilical incision, Group A), or the intervention arm (Pfannenstiel incision, Group B) a day before surgery. The trial executors receive randomly generated treatment allocations within sealed opaque envelopes. Afterwards, medical staff will personally inform the expert surgeon as to which treatment group the patient has been randomized. To avoid any potential prediction of group allocation, information on the block length will be kept away from the study site.

After the operation, the periumbilical and hypogastric regions will be covered with a double wound dressing until discharge. During this period, the severity of pain, rescue analgesic, and PEFR will be evaluated by anesthesiologists blinded to the incision type. Participants will also be blinded to the incision type. If blinding could affect the patient’s treatment, for example in the case of a medical emergency or serious medical condition, then the participant/assessor will be unblinded. After POD 4, a nephrologist who is unaware of the study will evaluate and document the primary and secondary endpoints of the trial. At the end of the trial, the data management center will receive all sealed envelopes and will check the accuracy of randomization numbers.

### Data management

All data will be collected and recorded in case report forms (CRFs) by an investigator before transfer to the data management center. To ensure accurate data collection, after each patient has been visited, the CRF will be completed by an investigator who did not evaluate the patient. All demographic and baseline clinical data, as well as primary and secondary outcome measures, will be recorded in the CRF. All data will be checked, and any missing data will be obtained from the trial database or from participants. To ensure patient confidentiality, the CRF for each patient will be given an anonymous allocation number. We will obtain permission to continue follow-up and data collection in the event of withdrawal from the study. The responsible investigator must review and sign all completed CRFs. Afterwards, data will be statistically analyzed by a statistician who is unaware of the allocated treatment.

### Statistical methods

#### Sample size

The null hypothesis is that Pfannenstiel incision is not superior to periumbilical incision in terms of the average number of days it takes the donor to return to normal physical activity in the 4 weeks after surgery. We chose 10 days difference in return to normal physical activity as the minimal clinically important difference, because a difference of less than 10 days is not valuable enough to decide the standard surgical method. Therefore, we used a difference of 10 days for sample size calculation. The standard deviation is expected to be 13 days, according to the results of El-Galley et al. [15], who compared days to return to work between periumbilical and Pfannenstiel incision groups. However, we chose “days to return to normal physical activity” as the primary endpoint rather than “days to return to work” because the nature of patients’ work and their capabilities are different, and some of our living donors may be unemployed or housekeepers. A one-sided significance level of α = 0.05, a power of 80%, and a sample size of 44 patients (22 per group) are required to apply a two-sample *t* test (calculations performed with AddPlan 6.0). To account for a 15% drop-out rate, 26 patients will be enrolled in each study arm (52 patients in total).

#### Statistical analysis

The primary endpoint is the number of days from surgery to return to normal physical activity. This will be assessed using the Katz ADL questionnaire. The primary analysis will test the following hypotheses:

H_0_ μ_Pfannenstiel_ ≥ μ_Periumbilical_

H_1_ μ_Pfannenstiel_ < μ_Periumbilical_,

where μ_Pfannenstiel_ and μ_Periumbilical_ are the mean number of days in the respective intervention groups. The primary endpoint will be compared between the intervention groups using a Student’s *t* test and a one-sided significance level of 5%. Additionally, a Mann-Whitney *U* test will be performed for sensitivity analysis. According to the intention-to-treat approach, all patients will be analyzed in the group to which they were randomized.

All secondary outcomes will be analyzed using appropriate summary measures depending on the distribution of the data and descriptive *p* values. Categorical data will be presented as absolute and relative frequencies. Continuous data will be presented as mean values with standard deviations and by median values with interquartile ranges, minimum values, and maximum values. Statistical analysis will be performed using SPSS version 24.0 (SPSS, Inc., Chicago, IL, USA). A statistical analysis plan will be developed by the principal investigator by the end of recruitment.

## Discussion

LDKT is one of the best solutions for organ shortage. Since living kidney donors give others a second chance to live, it is very important that they undergo the safest possible surgical procedure with the lowest risk and complication rate. Although both HALDN procedures (periumbilical and Pfannenstiel) are accepted surgical approaches [22–25], these two different incision methods have not been compared in an RCT. This RCT was initiated to compare the outcome of periumbilical and Pfannenstiel incisions.

Only one RCT has compared HALDN using a periumbilical incision with pure laparoscopic donor nephrectomy using a Pfannenstiel incision for graft extraction [26]. The authors compared operation time, warm ischemia time, complications, and quality of life between the two groups, but they did not report the time it took for the donor to return to work/normal physical activity or wound appearance. This RCT also compared two different methods (pure laparoscopy versus HALDN) as well as two types of incision. There are also some published non-randomized or retrospective studies that compare periumbilical and Pfannenstiel incisions for hand assistance and/or kidney removal in HALDN or laparoscopic donor nephrectomy [15–18]. However, although El-Galley et al. [15] reported the time to return to normal activity and work, none of these studies evaluated the donor’s comfort and satisfaction; they mainly compared the occurrence of complications and length of hospital stay after the two procedures.

The HAPERPACT trial will be the first RCT to evaluate the impact of periumbilical and Pfannenstiel incisions on donor recovery. We will compare the time it takes for the donor to return to normal physical activity after the two different procedures. This will indicate patient satisfaction and comfort, as well as the influence on economy and burden of disease.

The secondary findings of this RCT will compare the intraoperative and postoperative results in living kidney donors. The HAPERPACT trial is novel in that it investigates all factors associated with donor satisfaction after nephrectomy, including postoperative pain, rescue analgesics, PEFR, physical activity (using IPAQ), length of hospital stay, time to return to work, patient satisfaction, and cosmetic score. To ensure that our findings are more encyclopedic, the quality of the transplanted graft and graft function in recipients will also be monitored in this trial.

One limitation of this RCT is that the primary endpoint cannot be evaluated blind. However, some early outcome measures will be evaluated double blindly (participant and assessor blinded) until discharge by covering both periumbilical and hypogastric areas using double wound dressing. Furthermore, to prevent bias in further assessments, all follow-up visits will be performed by a nephrologist who is unaware of the study. Another limitation is that the primary outcome of this RCT is a subjective, self-maintenance measure. However, results of the Katz ADL self-administered questionnaire are known to be valid and reliable [27]. In addition to the Katz ADL questionnaire, we will also use the established IPAQ-SF [28], which will be completed by the investigator before and after surgery. There is no data monitoring process in the HAPERPACT trial, because the methods used are both standard surgical procedures for living donor nephrectomy and also because the biggest part of documentation in this trial is the standard clinical follow-up of the patients. Therefore, a data monitoring committee is dispensable in this trial.

In summary, published retrospective and non-randomized prospective studies have not clarified the advantages and disadvantages of periumbilical versus Pfannenstiel incisions in HALDN. The HAPERPACT trial will be the first RCT to compare the outcome of HALDN using two different types of incision. The comprehensive findings of this study may help to decide the optimal nephrectomy procedure for earlier return to normal physical activity, comfort, and satisfaction in donors as well as graft function in recipients.

### Trial status

The HAPERPACT trial is currently recruiting participants.

## Additional file

Additional file 1: SPIRIT checklist. (DOC 123 kb)

## Abbreviations

ADL: Activities of daily living
CRF: Case report form
DGF: Delayed graft function
GFR: Glomerular filtration rate
HALDN: Hand-assisted laparoscopic living donor nephrectomy
IPAQ-SF: International Physical Activity Questionnaire-Short Form
LDKT: Living donor kidney transplantation
PEFR: Peak expiratory flow rate
PNF: Primary non-function
POD: Postoperative day
POW: Postoperative week
RCT: Randomized controlled trial
SAE: Serious adverse event
VAS: Visual analog scale
